# Challenges and knowledge gaps in corrosion and materials degradation of bioreactors for waste feedstocks

**DOI:** 10.1007/s43939-026-00698-0

**Published:** 2026-06-05

**Authors:** Maria Ramos-Suarez, Yue Zhang, Julian Wharton, Sonia Heaven

**Affiliations:** Faculty of Engineering and Physical Sciences, University of Southampton, University Road, Southampton, SO17 1BJ UK

**Keywords:** Environmental biotechnology, Industrial biotechnology, Corrosion, Abrasion, Bioreactor material, Fermentation, Microbially-influenced corrosion

## Abstract

Industrial and environmental biotechnologies (IB/EnvB) are able to transform wastes and agro-industrial side-streams into bio-based products such as biomethane, biohydrogen, volatile fatty acids, lactic acid, succinic acid, ethanol, butanol, biosurfactants, enzymes and polyhydroxyalkanoates. Common corrosion drivers in the microbially-mediated production of these products are water, chlorides, hydrogen sulphide, acids, alcohols, biofilms, and concentration gradients or ‘dead’ zones within the bioreactor. The demand for cheaper bioreactor vessels, combined with the increasing use of these more challenging feedstocks, creates uncertainty about the durability of bioreactor materials. Current structural materials such as stainless steel, coated metals and composites offer good resistance to a wide range of exposure conditions; but may be more vulnerable to microbially-influenced corrosion and abrasion in novel bioprocesses utilising waste-type feedstocks. Existing standard test methods are insufficient, as they do not adequately characterise the potential for bioreactor materials degradation in realistic IB/EnvB conditions under long-term operation with combined corrosion-abrasion phenomena. Future research must prioritise identification and application of suitable testing strategies to ensure that the bioreactor manufacturing industry continues to provide reliable, cost-effective bioprocessing infrastructure for the new sustainable bio-economy.

**Graphical abstract:**

**Figure:**
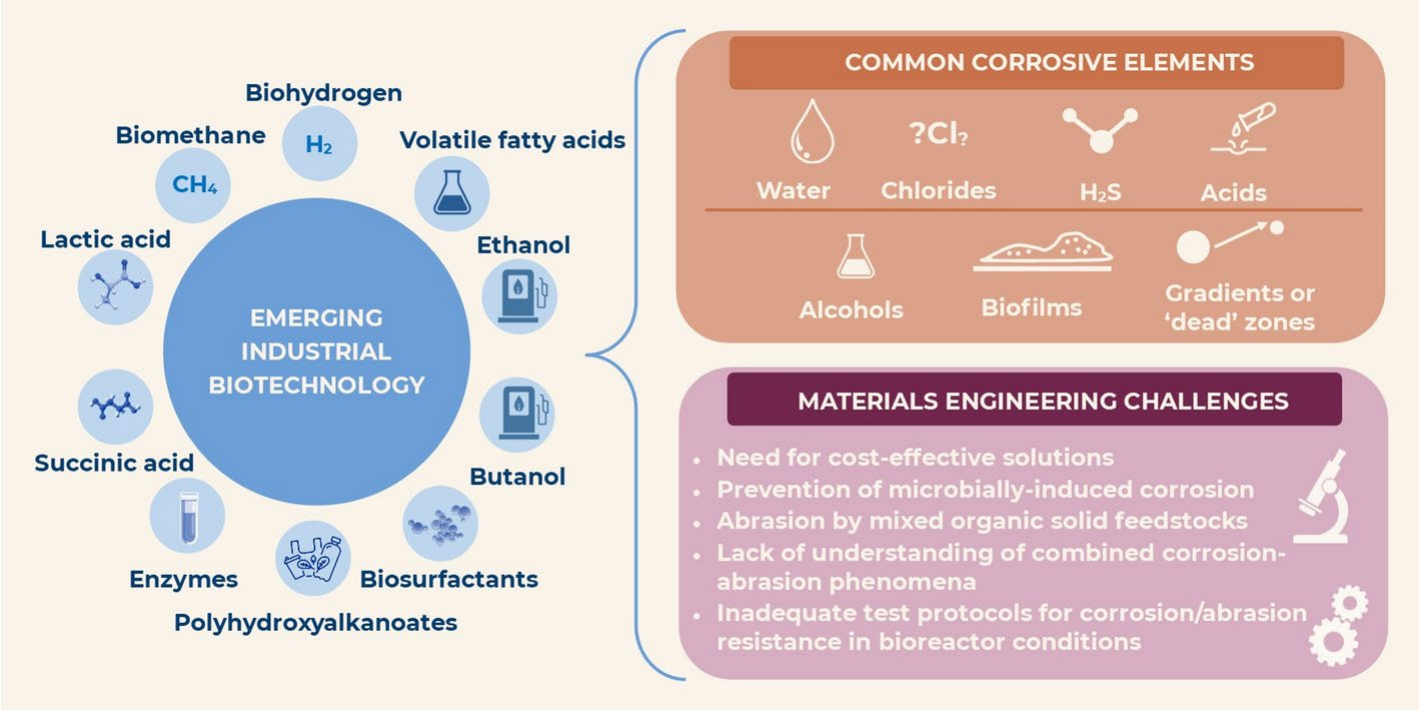

## Introduction

Climate change and other environmental challenges are driving the transition towards a bio-based economy focused on replacing products derived from finite resources, such as fossil hydrocarbons and mineral nutrients, with renewable and more sustainable alternatives [1]. One key element of this, known as ‘white’ or industrial biotechnology (IB) produces clean energy, chemicals, plastics, enzymes and other materials from renewable biological sources [2]. The importance of the IB sector is clearly reflected its global market size, currently valued at US$ 304 bn (2024) and predicted to reach US$ 626 bn by 2032 [3]. In the European Union, bio-based chemical production (excluding biofuels) accounts for only 3% of total chemical production, but could expand to a total of 156 Mtonne/year if fossil hydrocarbon-derived chemicals are replaced [4]. The significance of bio-based products is recognised and targeted to grow by policymakers in Europe [4], the UK [5] and USA [6]. In addition to economic drivers, development of the bio-based economy will help to achieve six of the United Nation’s 17 Sustainable Development Goals (SDGs), namely: affordable and clean energy; decent work and economic growth; industry, innovation and infrastructure; sustainable cities and communities; responsible consumption and production; and climate action [5, 7].

In contrast, Environmental Biotechnology (EnvB) focuses on environmental protection and bioremediation [8, 9], addressing SDGs on clean water and sanitation, life below water, life on land, and also good health and well-being. It is arguably the largest IB sector globally according to several measures, including volumes of material processed and number of customers served and staff employed. EnvB encompasses biotechnologies such as wastewater treatment and anaerobic digestion (AD) which break down or recycle organic materials to minimise their impacts on the environment [8]. These technologies lie at the heart of many IB systems from biochemicals and biomaterials production to biorefineries, and the European Leadership Group on Circular Bioeconomy recognises EnvB as an “essential foundation” of the bioeconomy [10].

EnvB deals with a wide range of organic residues, such as wastewater biosolids, municipal solid waste, food waste, agro-industrial by-products and sidestreams, livestock slurries and manures, which are available in large volumes but have challenging characteristics in terms of processing. There has been some debate whether EnvB must primarily be directed towards environmental protection or remediation, and only secondarily to generation of value-added products where this offsets the cost of treatment; or whether the mere use of a waste or by-product stream means a process should be regarded as EnvB rather than IB [3]. In practice, however, the shift to a circular bio-economy will mean a huge increase in demand for organic materials as feedstocks for resource recovery and product generation. It is likely therefore that any distinctions between IB and EnvB will reduce, with higher-quality materials being reserved for more demanding areas like food and pharmaceutical production and materials with challenging properties being increasingly in demand for bulk chemical production. This transition is essential for a resource-efficient economy, and IB/EnvB offers great potential to reduce waste, generate employment opportunities, decrease CO₂ emissions, cut energy costs, and drive the development of new and more sustainable industries [5].

**Fig. 1:**
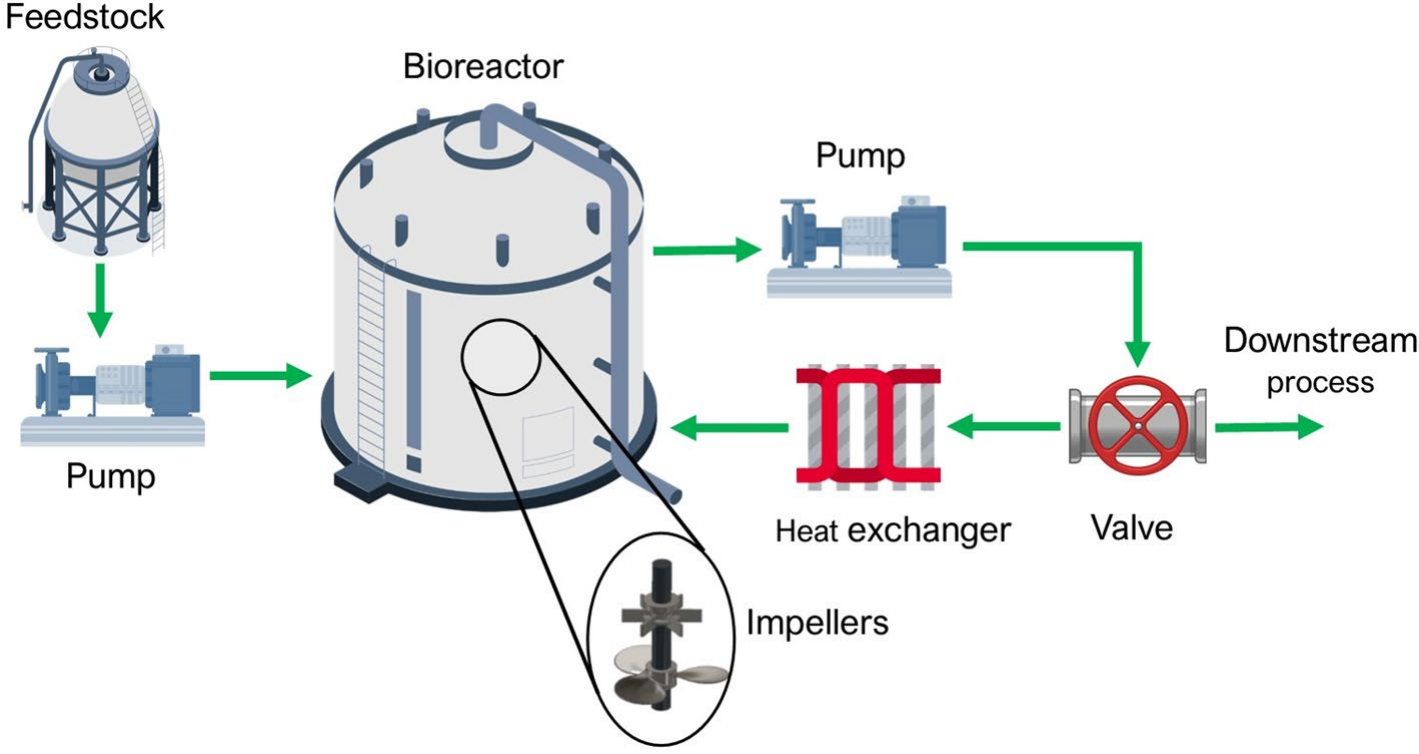
Example of bioreactor and ancillary equipment configuration

The microbial transformations for product generation in IB/EnvB take place in various bioreactor types (stirred tank, air lift reactor, fixed bed, fluidised bed, membrane reactor, etc.) and operating modes (batch, fed-batch, and continuous) [11]. In any such process, bioreactors must be designed for optimal heat, gas and mass transfer as well as providing the appropriate environment for microbes to carry out their desired functions. Bioreactor sizes and materials vary depending on application. In large-scale applications (250 m^3^ working volume and above), metal is the most common material of construction for bioreactor vessels. Corrosion and biodegradation of bioreactor internal walls and infrastructure, such as pipelines and pumps, can occur progressively during extended operation, with the rate of degradation influenced by the specific process conditions and operating environment. A simplified example of a bioreactor and ancillary equipment configuration is shown in Fig. 1. This is a typical configuration for EnvB applications such as AD, although internal mixing may not be required if recirculation rates are high. Corrosion failures in such systems, particularly reactor vessel leaks caused by pitting corrosion, can lead to serious impacts on the environment and human health, as well as causing significant financial loss [12]. The design life of a bioreactor is commonly taken as 10–20 years [13], although much longer periods in service are also common in practice. Bioreactors and ancillary equipment often represent a major capital investment: for example, in AD of food wastes, bioreactors and their ancillary equipment typically represent 35–49% of the total capital cost of a plant [14]. In a bio-acetone-butanol-acetone production plant, the bioreactor itself is 17–20% of the total capital cost, including the product recovery stage, and 51% of the capital cost of the fermentation section alone [15]. While some auxiliary equipment may be exposed to more corrosive environments (for example, acid/base streams for pH correction), a bioreactor failure can result in shutdown and the disruption of a tightly balanced metabolic activity, which may take many weeks to restore. It is therefore essential to better understand the degradation mechanisms in bioreactors during extended service, and to select materials and coatings with appropriate properties to ensure long-term resilience and process efficiency.

The bioreactor manufacturing industry has extensive knowledge and experience of existing structural materials: but growth in IB/EnvB applications coupled with the relatively low value of bulk commodity chemicals means there is a push to develop new and cheaper tank systems. The compatibility of existing and new operating conditions with novel bioreactor materials and coatings is unknown. At the same time IB/EnvB feedstocks and bioreactor contents contain a rich population of microorganisms, but the implications of this for microbially-influenced corrosion (MIC) are still relatively understudied. These challenges are often compounded by the inaccessibility of the reactor interior for direct inspection, which means reactors may operate for years or even decades without any knowledge of the conditions of materials and coatings.

Apart from corrosion and MIC, another surface degradation mechanism contributing to fermenter/bioreactor failure is abrasive wear. The abrasive properties of new IB/EnvB feedstocks differ from those that have traditionally been used in IB, especially for food or pharmaceutical production. As the medium flows through or is agitated within the bioreactor, abrasive particles can cause wear to the internal surfaces and to moving components such as impellers, potentially compromising mechanical integrity over time.

This review provides a first targeted effort to identify knowledge gaps in bioreactor materials selection and testing for IB/EnvB applications with feedstocks derived from waste and agro-industrial side streams (waste-related feedstocks). It identifies the top 10 most researched and value-added bio-based products, and the key operating conditions that may influence bioreactor material deterioration. Additionally, it critically reviews current material testing methodologies to determine their suitability under emerging feedstock compositions and process environments.

## Bioprocessing of waste substrates - overview

**Fig. 2:**
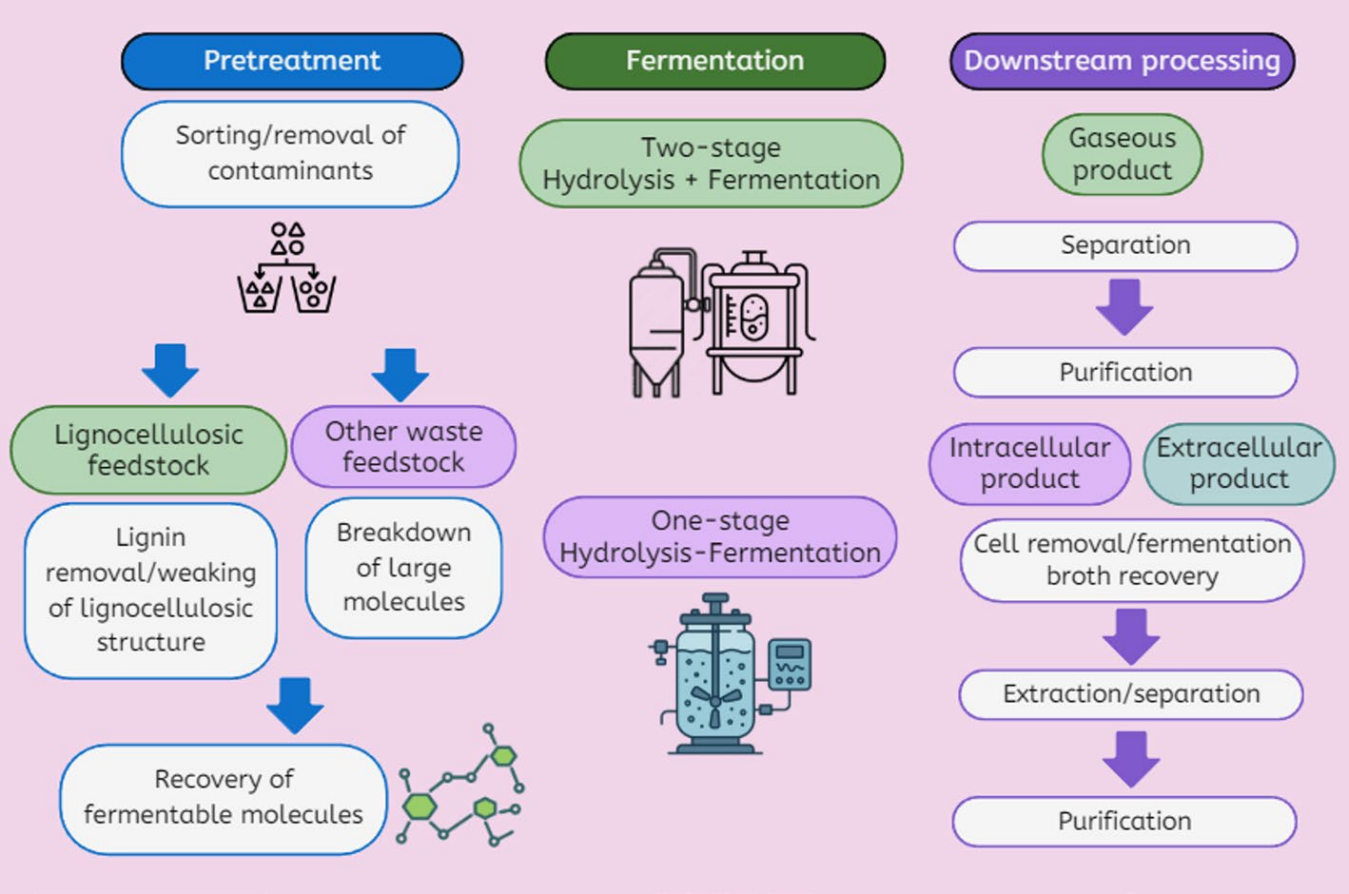
Industrial biotechnology process overview in stages using waste or lignocellulosic substrate

An overview of a typical waste-based bioprocess, including pre-treatment, fermentation and downstream processing, is shown in Fig. 2. Waste-related substrates may require pre-treatment to remove contaminants, reduce particle size, and homogenise the material. Following this, further pre-treatment might be required to improve conversion into valuable products. This may involve the breakdown of large molecules or, in the case of lignocellulosic wastes, the removal or ‘weakening’ of the lignocellulose. Pre-treatments can be mechanical, physical, biological, chemical or physico-chemical [16, 17]. Such pre-treatments will affect feedstock characteristics and process conditions in the main bioreactor, but may also represent the most stringent exposure conditions for vessels and auxiliary equipment in the overall process chain.

The fermentation stage can be classed as single-strain or microbial mixed culture (MMC). Normally single-strain fermentations require sterilisation, in contrast with MMC processes which can often be operated under non-sterile conditions. Bioprocesses which require feedstock hydrolysis may be classified as one or two-stage hydrolysis-fermentation, with the latter needing two reactors with different operational conditions. Depending on liquid content, fermentations can also be classed as solid-state fermentation (SSF) or submerged fermentation (SmF). SSF takes place in the absence (or near-absence) of free water [18]. SmF takes place in a free-moving liquid medium, and is used in the majority of large-scale commercial settings. SmF is dominant in current industrial practice due to better control of fermentation conditions, but SSF is sometimes preferred for solid substrates such as agro-industrial lignocellulosic wastes due to higher productivity, higher product concentrations, low energy consumption, low water consumption and better aeration [19].

Fermenters and hydrolysis tanks require auxiliary equipment for operation, such as pumps, agitators, feed tanks, heaters and control systems (sensors). Depending on the process configuration and design, these elements may be exposed to aging factors similar to those in the main reactor. While some ancillary equipment can be replaced without disturbing process continuity, repairing and replacing bioreactors generally requires complete shutdown, sometimes including disposal of the reactor contents, and is thus disruptive and expensive.

## Surface degradation mechanisms

All bioreactor materials undergo aging and degradation, driven by mechanical stresses (such as fatigue and partial collapse/buckling) or environmental factors (moisture, temperature, UV radiation, chemicals, and biological agents), both of which can compromise structural integrity and performance. Aging leads to structural and chemical changes, and a reduction in molecular weight. In this work, the focus is primarily on environmental degradation, as the main concern is material compatibility with operating conditions, bioreactor contents and the effects of microbial activity.

Corrosion is a surface deterioration process associated with chemical or electrochemical interactions with the adjacent environment [20], in this instance the IB/EnvB media. Corrosion most commonly affects metals such as iron/steel, aluminium, copper and zinc [20], leading to the formation of layers such as oxides, sulphides or carbonates, and involves the formation of anodes and cathodes within a system [13]. This process can manifest as uniform corrosion, which may result in a general thinning of metal plates; or as pitting corrosion, which generates localized holes (including through-plate perforations) [21]. Crevice corrosion may occur due to the formation of micro-environments, linked to parts of the metallic surface being shielded from adjacent conditions (e.g., gaps between joined plates and non-metallic deposits), while galvanic corrosion results from the coupling between dissimilar metals [21]. Corrosion often interacts with other aging mechanisms, contributing to complex phenomena such as erosion-corrosion, stress-corrosion cracking (SCC), hydrogen embrittlement and corrosion fatigue [21].

Microbiologically-influenced corrosion (MIC), also known as biocorrosion, is the direct or indirect corrosion of metals induced by microorganisms, typically bacteria, which are present in the medium in contact with the metal surface. MIC is most detrimental when microbes have formed an aggregation called biofilm, which attaches to the metal surface [22]. This is because biofilms induce the formation of chemical concentration cells, causing pH and chemical gradients [23]. Biofilms are composed of embedded microbial cells, extracellular polymeric substances (EPS) and metabolites (organic and inorganic) [24]. MIC can take place through the following mechanisms: oxidation-reduction reactions [25]; production of corrosive compounds such as acids [25] or reduced sulphur compounds [26]; production of enzymes [25]; creation of chemical gradients and trapping of metal ions [25]; removal of corrosion-inhibitory substances [26]; and utilization of protective coatings as a carbon source [26]. Despite the presence of microorganisms in IB/EnvB processes, MIC is not a widely studied phenomenon in these applications. Most of the literature on MIC focuses on the oil & gas industry [27], marine environments [28], and water and wastewater pipelines [29, 30]. Microbes that contribute to MIC include sulphate-reducing bacteria (SRB), iron/manganese oxidising bacteria, iron-reducing bacteria, sulphur-oxidising bacteria, and organisms that produce organic acids and slimes [31]. The majority of MIC studies focus on SRB [22] or nitrate-reducing bacteria (NRB) [24] in the oil & gas industry or marine environments. SRB reduce sulphate ions to sulphide, which is highly corrosive to many metals [26]. NRB such as *Pseudomonas aeruginosa* reduce nitrate to ammonia under aerobic conditions, which can cause corrosion in steels [24]. SRB are of more concern than NRB in fermentation systems, as most fermentations take place under anaerobic conditions. However, many other types of microorganisms can cause MIC. For example, members of the genus *Thiobacillus* cause corrosion of metal, concrete or limestone by oxidising sulphur or sulphide to form sulphuric acid [26]. *Gallionella*, *Sphaerotilus* and *Pseudomonas* also cause corrosion of steels and stainless steels under aerobic conditions [23]. On the other hand, fungi can produce acids which can corrode materials as well as depleting oxygen, thus generating perfect anaerobic conditions for SBR to thrive under the fungal mats [26]. In the oil & gas sector, internal pipeline MIC typically develops in saline, sulphate-bearing produced waters under hydrocarbon carryover, often at elevated temperature/pressure and high wall-shear; control strategies rely on pigging and periodic biocide dosing [32]. SRB-dominated biofilms, sulphide formation and localized, deposit-shielded corrosion are frequent concerns. In contrast, anaerobic digesters and fermenters operate as living bioprocesses with abundant nutrients and a dense microbial population to convert organic carbon-based feedstocks. Thus, biocide use is not feasible, and corrosion risk is tied to microbial community dynamics (SRB vs. methanogens), headspace/intermittent wetting, solids loading (nutrients) and shear regimes that differ from those in pipelines. In MMC systems, nitrate addition may help to suppress SRB activity [26]; however, this introduces additional operating costs and may be detrimental in nutrient-limited fermentations. Super-hydrophobic surfaces have shown promise in preventing biofilm formation by maintaining a gas layer in submerged conditions, but they are highly vulnerable to physical damage and thus unsuitable for bioreactors handling waste-related feedstocks [31]. Surface scrubbing is another common practice to mitigate MIC in pipelines, but this technique may not be compatible with typical bioreactor operation and may introduce issues related to erosion. Interestingly, some microbial communities and biofilms may act as corrosion inhibitors by forming protective barriers against the bulk solution [23] or producing anti-corrosive metabolites; certain strains have shown potential as biocorrosion inhibitors in non-bioreactor environments [31].

In contrast to metals, polymers, are liable to deterioration caused by chemicals, solar radiation, temperature, moisture and microbial growth [33, 34]. While microbes typically cannot digest high molecular weight polymers, biofilms can form on polymer surfaces, particularly when the surface becomes rough [33], leading to a shortening of polymer chains [34]. Degradation mechanisms in polymers include chain scission, oxidation, erosion [33], formation of macro-radicals, changes in end-groups, and formation of peroxides or carbonyl species [35]. Other physical changes that polymers may undergo include melting, sublimation, evaporation, crystallisation, cross-linking, charring and water absorption [35]. This will induce changes in colour, mass, density and shape, and eventually the development of cracks and voids which lead to failure of the material for its intended purpose [35]. Thermoplastic polymers can be dissolved by certain solvents, or the solvents may be absorbed causing polymer plasticisation [20]. For example, PLA can be dissolved by acetonitrile, toluene and, to some extent, ethanol in a process known as aminolysis [36]. Alcohols and polyols are effectively used to depolymerise PET plastics [37].

Abrasion is a mechanical wear process caused by the interaction of hard particles or surfaces with softer material. All materials are vulnerable to abrasion, which generates grooves or indents (two-body or three-body abrasion, respectively) or fractures/cracks [38]. This can accelerate degradation and corrosion, particularly MIC, as the resulting crevices provide ideal locations for microbial biofilm formation. Abrasion typically refers to *dry* environments (air or gaseous media), and the term *slurry-erosion* describes similar wear caused by the combined action of liquid and solid abrasive particles [39]. Here, the term ‘abrasion’ is used for both liquid and gaseous media.

## Materials in bioreactor construction for industrial and environmental biotechnology

The selection of materials for a bioreactor and its ancillary equipment must align with process conditions to ensure optimal performance and lifespan. A summary of materials suitability, characteristics and cost implications is shown in Table 1.

The main materials used in IB/EnvB applications are iron-based alloys, such as stainless [40–48] or carbon steels [49–52]. Resistance to corrosion by SS is due to the formation of a passive oxide film (passivation), which breaks down when exposed to oxidising salts such as chlorides and some organic acids [13]. This film is extremely thin, at several nanometres (depending on the environmental conditions), and it forms and reforms until it cannot be replenished by gaseous or dissolved oxygen [13]. Nickel, molybdenum, titanium, manganese, copper, titanium and silicon are added to SS alloys to improve resistance to various types of corrosion [13]. The two most common grades of SS are 304 and 316. 304 SS contains 18% chromium and 8% nickel [53]. 316 also contains molybdenum (2–3%), making it more corrosion resistant [53]; however, it can be more susceptible to oxidising acids such as nitric acid [53]. For fermentation applications, the most compatible alloys are 316 SS, Inconel 600, Hastelloy C-276, Incoloy 825 and Carpenter 20CB-3 [13].

Despite the inherent resistance of SS alloys, corrosion can still occur under certain conditions. To enhance durability, some manufacturers supply tanks with corrosion resistant coatings such as enamel, also known as glass-fused-to-steel (GFtS) [40, 44–48, 54–57] and epoxy [40, 44–46, 48, 54, 55, 58]. These coatings are widely used in the water/wastewater treatment, chemical and agricultural industries. GFtS offers excellent corrosion resistance to both acidic and alkaline environments, with the exception of hydrofluoric acid (HF) and concentrated phosphoric acid (H_3_PO_4_) [59]. The pH range resistance of GFtS is dependent on the number of coats and the application method [60]. Glass linings have high durability and abrasion resistance, but can be costly and may contain pinholes formed during application [59]. Epoxy coatings, in contrast, are not only used as protective linings, but can also serve as repair patches where welding is not recommended (e.g. near explosive atmospheres); but they have lower abrasion resistance and less is known about their durability or operational lifetimes [59]. Epoxy-coated steel tanks can tolerate pH ranges of 3–13, and have high corrosion and impact resistance, with a hardness of 2.5 in the Mohs scale [61]. Chemical compatibility and physical characteristics of epoxy resins are dependent on resin base (e.g. bisphenol A, bisphenol F, and Phenolic Novalac) and their co-reactant or hardener (e.g. polyamide, amidoamine or aromatic amine), with Novalac epoxies having the highest chemical resistance [62].

Galvanised steel, formed by immersing steel in molten zinc to create a protective layer, is also widely available in the market for water storage [54, 63, 64], but is not common in IB/EnvB applications. Galvanised steel has a narrow tolerable pH range (6–12) [65], forming white rust under acidic conditions [66]. Suppliers to the petrochemical industry offer steel coatings made with polyurethane, polyamine cured phenolic, epoxy, epoxy zinc phosphate and polyamine cured epoxy, among others [67]. Polyurethane coating is reported as ideal for dilute acid and industrial chemicals [58].

After metal, plastic is the most common material used for tank manufacture. Examples of plastics used for storage/process solutions include high density polyethylene (HDPE) and polypropylene (PP) [68]. Other polymers used include polyurea, hydrophobic polymer, vinyl ester, and epoxy novolacs [69].

Another alternative is the use of composite materials such as glass fibre-reinforced plastic (GFP). GFP has some advantages compared to steel, particularly its low weight, high tensile strength, non-conductive properties and superior corrosion resistance [70]. GFP tanks are commonly used in the food [68, 71], potable water [44, 72–74] and chemical industries [75]. One variant of GFP is glass flake epoxy [69]. However, GFP is not widely used in IB/EnvB applications, except for a patent on GFP tanks for biogas production [76]. GFP can also be applied as a lining for concrete or steel tanks used for water storage [77]. On the other hand, some manufacturers claim SS offers better performance than GFP for water storage, citing benefits such as durability, corrosion resistance, hygienic properties (non-porous) and recyclability [78].

In the wastewater treatment sector, digesters are often made of concrete as it presents some advantages over steel, such as better corrosion resistance, better insulation characteristics, option to re-drill and extended lifetime [79]. Concrete digesters are also sometimes used in agricultural applications [80]. Concrete used for bioreactor construction is generally reinforced with metal, typically steel [81]. Chlorides can ingress concrete structures and depassivate the reinforcement, causing cracking of the concrete [81]. CO_2_ also causes carbonation of the concrete, making conditions less alkaline and reducing the protection of the steel against corrosion [81]. Linings such as epoxy, polyurethane and polyurea can be applied to concrete digesters to improve their durability [82].

Bioreactors can also be made of glass and ceramics. Borosilicate glass is the most commonly-used material for glass-based bioreactors as it is particularly unreactive, but its fragility means the size of such reactors is usually limited to pilot-scale applications [83]. If cracks or scratches are present in silicate glasses, water and water vapour can accelerate the crack growth velocity [84]. Ceramics (oxides, carbides, nitrides or sulphides) are chemically stable and wear-resistant [84], but brittle and vulnerable to thermal shock [83]. Ceramics are also used as membranes in membrane bioreactors [85]. Since these membranes tend to foul and need replacement, they can be made of low-cost materials such as clay, calcium carbonate, potato starch, almond shell and chamotte [85].

Coated textiles offer some advantages due to their flexible geometry. They can be easily folded and transported and are relatively low cost. The textiles must be water and gas-proof. Although they can be highly resistance to biodegradation, they are susceptible to physical damage (piercing and cutting) [83]. Materials used include polyvinyl chloride (PVC)-coated textiles such as polyester [83]. PVC-polyester, however, exhibits poor compatibility with solvents and low thermal stability [83]. Alternatively, all-polyamide composite coated textiles (APCT) offer better durability [83]. While manufacturers are working on new materials and coatings, there is limited historical data on their performance in biotechnological applications.

**Table 1:** Summary of bioreactor material suitability, disadvantages and cost

Material	Suitability	Characteristics	Cost implications (for year 2025)
Stainless steel	Resistant to most corrosive elements. Better for aerobic conditions. Ideal for processes which require sterilisation	Thermally stable. Structural material	SS 304 €2,300-2,380 per tonne and SS 316 €3,600-3,680 per tonne [86]
Carbon Steel	Typically requires protective coating	Thermally stable. Structural material	€561–643 per tonne [87]
Enamel (glass fused to steel)	High corrosion resistance. Suitable for wide range of pHs (1–14). High abrasion resistance	Thermally stable. UV resistant	Highly dependent on coating method and supplier
Epoxy	High corrosion and impact resistance. Suitable for wide range of pHs (3–13). Heat resistant. Suitable for anaerobic digestion	Prompt to chalking and discolouring. Brittle	€4,370-4,710 per tonne [88]
Galvanised steel	Not common in biotechnological applications. Ideal for water storage or hygienic applications. Great resistance to corrosion and wear. pH range of 6–12	Greater chloride resistance than untreated steel. Poor resistance to strong alkalis and acids	€739–813 per tonne of galvanised steel [89] or €2,570–2884 per tonne of zinc [90]
Concrete	Better corrosion resistance than steel	Easy to drill	€130–150 per m^3^ [91]
Borosilicate glass	High corrosion resistance. Only suitable for small scales	Thermally stable. Fragile	Typically sold as part of complex expensive equipment
Ceramic	High corrosion resistance. Suitable for high salinity	Brittle and subject to thermal shock. Porous material	€910 per thousand bricks [92]
high density polyethylene (HDPE)	High chemical and corrosion resistance. Suitable for salty environments. Abrasion resistant	Lightweight. Thermally unstable from 120 °C. Subject to aging by UV	€962–966 per tonne [93]
polypropylene (PP)	High chemical and corrosion resistance. Suitable for salty environments. Excellent impact resistance	Water absorbent. Thermally unstable	€966-1,005 per tonne [93]
glass fibre-reinforced plastic (GFP)	High corrosion resistance	Low weight, high tensile strength, non-conductive	Highly depends on supplier and design
Coated textiles	Insufficient historical data	Flexible. Subject to piercing and cutting	Depends on material and supplier

## Top bio-based commodity chemicals

This section identifies the top 10 commodity chemicals which can be derived from waste-related feedstocks. Such feedstocks are unlikely to be utilised directly for food or pharmaceutical products, where standards for corrosion and materials testing are already in place [94]; and food and pharmaceutical applications are therefore not included in the scope. More exhaustive lists of bio-based products from renewable sources can be found in the literature [95–98].

Rankings of commercial significance are typically based on market size of the final product [99] or product application [4]. Given the wide variety of products that can be derived from these processes, this discussion focuses on the top 10 microbially-derived compounds, or groups of compounds which are similar in nature, selected according to two factors: research interest over the past two decades, and market size.

**Fig. 3:**
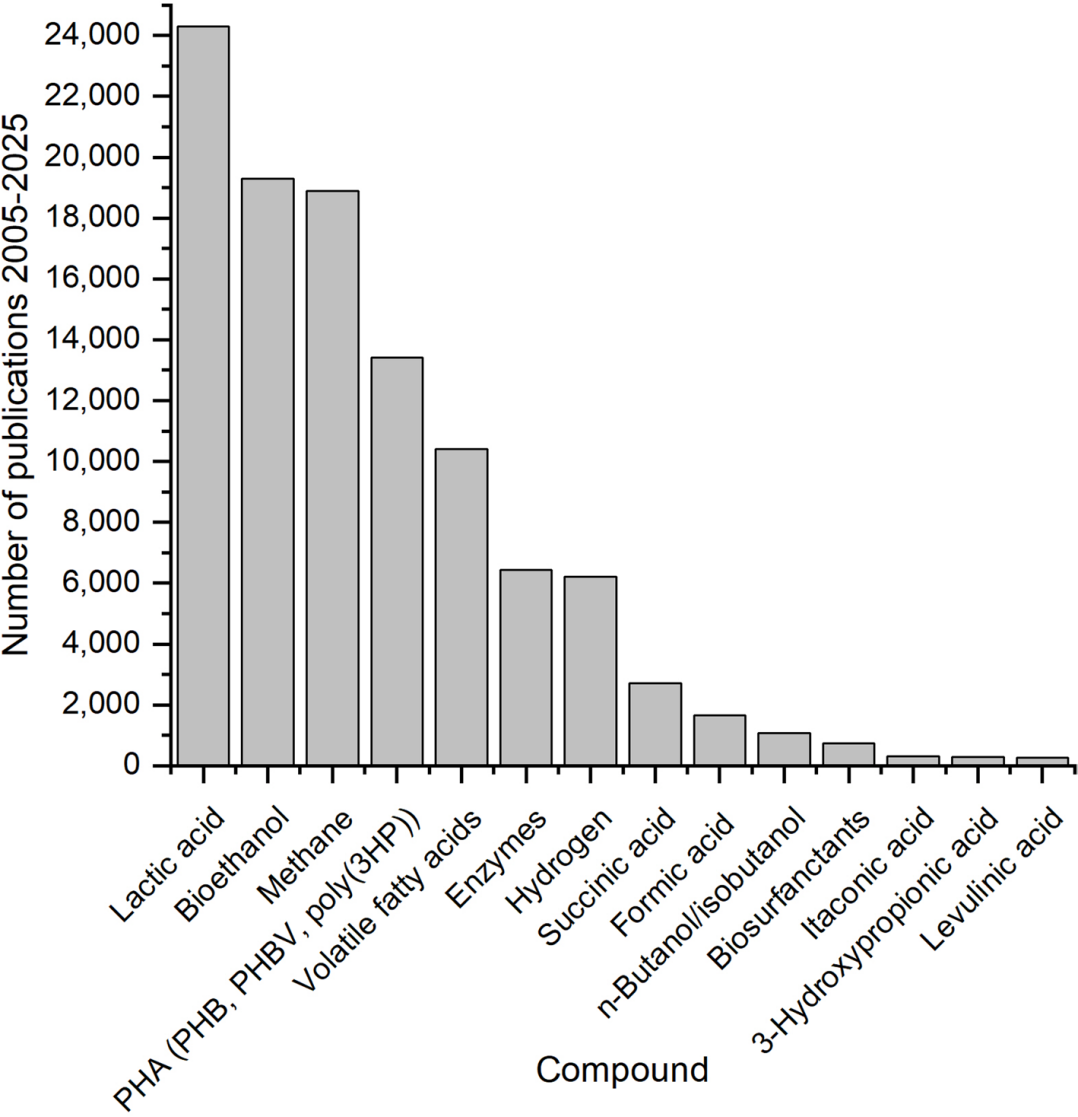
Number of publications on relevant biological processes in 2005–2025

Figure 3 shows the number of publications in the 2005–2025 period for individual bio-based products or groups of products. Compounds with less than 200 results have not been included here, but the full list can be found in Appendix 11., Table 5. Lactic acid, bioethanol, biomethane, Polyhydroxyalkanoates (PHA), enzymes, biohydrogen, succinic acid, formic acid, n-butanol/isobutanol, biosurfactants, and itaconic acid are among the most researched bio-based products. In the case of PHA, polyhydroxybutyrate (PHB) alone represents 32% of the outcome.

Special attention is given to bio-ethylene, one of the main bulk chemicals used for the synthesis of polymers, which can be produced biologically [100] but is more commonly synthesized through the catalytic dehydration of bioethanol [101]. The production of bio-based polyethylene terephthalate (PET) from bioethanol follows a similar route [102]. Consequently, bio-ethylene and bio-polyethylene (Bio-PE) and Bio-PET are categorized here as a derivative of bioethanol. Iso-butanol is the intermediate for bio-polypropylene (Bio-PP) production, following various chemical transformations [102], therefore iso-butanol was the bio-based product investigated. A similar approach was followed for polylactic acid (PLA) which is derived chemically or biologically from lactic acid [103]. Likewise, 2,5-furandicarboxylic acid (FDCA) can be chemically synthesised from sugars, therefore the research on fermentation is currently limited (20 publications).

Table 2 shows the market size, price, compound annual growth rate (CAGR) and global production for each bio-based product. Although the bio-based hydrogen market size is relatively small (US$ 78.6 million in 2025) [104], it is a by-product of VFA production and is likely to be valorised. About 500 billion m^3^ of hydrogen are produced yearly, 95% of which is fossil hydrocarbon-derived [105]. Based on economic factors relevant bio-based products appear to be hydrogen, bioethanol, enzymes, methane, VFA (combining market sizes for acetic, propionic and butyric acid), lactic acid, n-butanol/iso-butanol, and biosurfactants. While formic acid fermentation is highly researched, it appears to have fewer economic drivers than butanol and biosurfactants. Formic acid can be considered a VFA, although it is typically a trace by-product in MMC acidogenic fermentation systems. Formic acid is predominantly manufactured via chemical synthesis from carbon monoxide [106] (petrochemical route), but it can also be sustainably produced through green chemistry routes from biomass or CO_2_ [107]. Despite PHA being in the top 10 most researched compounds, PHA market size is among the smallest; however, PHA has a high CAGR of 29.2% [108]. Estimates of PHA market size vary greatly (US$ 31 to 715 million) [108–111]. Polybutylene succinate (PBS) market size is notably higher, with an estimated value of US$ 399 million [112]. PHA and PBS fall in the category of ‘bioplastics’, for which market size is estimated at US$ 16.9 billion (2024) [113]. However, definitions of bioplastic are broad and can include fossil-based plastics that are biodegradable [114]. Both PHA and PBS are bio-based biodegradable plastics, but PBS is chemically derived from succinic acid and 1,4-butanediol, the latter mostly from fossil sources [115]. Therefore, PBS production has been excluded in this work. While succinic acid has a relatively low market size, the main alternative to petrochemical synthesis is microbial fermentation [95].

**Table 2:** Economic data for different bio-based products

Bio-based product	Product application	Market size (million US$)	CAGR	Global production (tonne/year or otherwise)
Hydrogen	Fuel for fuel-cell vehicles, heat and power generation and raw material for ammonia production [105]	262,130 (2024) [116]	7.8% (2024–2034) [116]	120·10^6^ [117]
Bioethanol	Predominantly used as fuel, but also in the formulation of disinfectants, personal care products, pharmaceuticals and chemicals [95]	87,540 (2024) [118]	7.4% (2025–2037) [118]	88.2·10^6 (1)^[119]
Enzymes	Ingredient or catalyst in food, leather, pulp and paper, detergent, pharmaceutical, textile, and cosmetic industries [95]	60,480 (2023) [120]	4.9% (2024–2030) [120]	1,235·10^6^ [121]
Methane (biogas)	Heat and electricity generation [18, 95]	58,230 (2022) [122]	5.2% (2023–2030) [122]	21,400 MW/year [123]
Acetic acid	Additive in food and cleaning products and precursor in chemical and plastic manufacturing [124]	16,130 (2023) [125]	6.8% (2024–2032) [125]	21.7·10^6^ [126]
Lactic acid	Additive in the food and beverage, cosmetic, chemical and pharmaceutical industries [95], and precursor in the production of PLA [18]	7,140 (2023) [127]	18.0% (2024–2030) [127]	1.5·10^6^ [128]
n-Butanol	Solvent, chemical precursor and biofuel [129]	5,420 (2024) [130]	4.1% (2024–2034) [130]	5.45·10^6^ [131]
Biosurfactants	Food, agricultural, petroleum, pharmaceutical and cosmetic industries [132]	4,410 (2023) [133]	5.4% (2024–2032) [133]	10·10^6^ [132]
Formic acid	-	2,110 (2023) [134]	9.5% (2024–2033) [134]	750·10^3^ [135]
Propionic acid	Food preservative and precursor in chemical manufacturing [136]	1,100 (2022) [137]	3.8% (2023–2030) [137]	463·10^3^ [138]
Iso-butanol	Solvent, chemical precursor and biofuel [139]	1,020 (2023) [140]	6.58% (2023–2033) [140]	-
Glucaric acid	-	1,028 (2023) [141]	6.5% (2024–2032) [141]	No information found
1,2-Propanediol	-	381 (2024) [142]	4.2% (2025–2031) [142]	1.36·10^6^ [143]
PBS	-	399 (2023) [112]	20.2% (2020–2032) [112]	-
Butyric acid	Additive and precursor in chemical, food, pharmaceutical, perfume, and animal feed industries [144]	317 (2022) [145]	9.2% (2022–2027) [145]	220·10^3^ [146]
Succinic acid	Synthesis of chemicals and polymers and additive in food and pharmaceutical industries [95, 147]	223 (2021) [148]	9.7% (2022–2030) [148]	70·10^3^ [149]
Itaconic acid	-	98 (2021) [150]	4.1% (2022–2030) [150]	41·10^3^ [151]
Levulinic acid	-	80 (2023) [152]	9.4% (2024–2030) [152]	No information found
PHA	Replacement of common plastics such as polyethylene, polypropylene, polystyrene, PVC and PET [99]	31 (2023) [108]	29.2% (2024–2032) [108]	45·10^3^ [153]

^1^ Unit conversion

## Material degradation agents in bioprocesses

### General process conditions

Commonly controlled process variables which may influence the degradation of bioreactor materials are temperature, pressure, dissolved oxygen, redox potential, nutrient/substrate concentrations and pH [11]. Optimum conditions depend on each specific bioprocess. The main conditions for the top 10 bioprocesses listed are summarised in Table 2. Bioreactors may require sterilisation, which might take place using steam at 120–122 °C (pressure 2.0–2.2 bar) [11] or chlorinated substances, potentially leading to corrosion in sensitised areas. The requirement for frequent sterilisation, however, is typically reduced in systems using waste-related feedstocks and MMC [13]. Chlorides are also present in fermentation media as KCl, NaCl, FeCl_3_, CaCl_2_, CuCl_2_ and CoCl_2_ [13]. HCl, considered highly corrosive, is also used as means of acidifying the medium when required [13]. In contrast, where acids are the target product, the addition of bases such as NaOH and Na_2_CO_3_ may be needed to reduce pH [11]. Alkali corrosion is less problematic for steels, but can negatively affect the chemical and mechanical properties of fibre-reinforced polymer composites [154]. In many cases, antifoam agents are also added to fermentations [11, 105]. Other frequently-used chemical additives include metal ions (0–0.6 g/L), metal monomers, metal oxides, vitamins (vitamin B1, biotin, nicotinic acid), ethylenediaminetetraacetic acid (EDTA, 0.2–0.3 mg/L)) and buffer solutions [105]. Nitrogen is also required for cell growth. Common nitrogen sources are urea, yeast extract, NaNO_3_, NH_4_NO_3_, peptone and meat extract.

The conditions presented in Table 2 are typical of those in the bulk fermentation medium. However, reactors may also contain ‘dead zones’ because of poor mixing. This can lead to areas where conditions are significantly different from measured values or setpoints. Regardless of mixing adequacy, reactor geometry and placement of inlets can also create gradients or areas of different conditions. For example, lower dissolved O_2_, lower pH, lower substrate concentration (if fed from the top), and higher pressure are commonly observed at the bottom of aerobic reactors [11]. These gradients can accelerate corrosion and aging of materials.

Some fermentation processes utilise support materials for cell immobilisation [11]. If the immobilisation material is suspended in the liquid (i.e., free-moving), for example as solid or porous particles, it may act as an abrasive agent against the bioreactor walls. Many methods of immobilisation involve the use of polymers such as alginate, agar, polyethylene glycol, etc. [105]. For adsorption immobilisation, matrices like activated carbon, biochar, alginate, sponges, agar, ceramics, polyurethane foam, lignocellulosic waste, microplastic, and carbon nanotubes are available [105]. For entrapment immobilisation, agar, alginate, cellulose, carrageenan, polyacrylamide, polyurethane, polyvinyl, and polypropylene may be used [105]. Other methods involve incorporating cells into a polymer matrix such as polystyrene, or PET [105]; or encapsulating in polyvinylidene fluoride [105]. The size of granules/immobilisers can vary, typically ranging from 1.5 to 30 mm or more [105]. There are no studies on the effect of these immobilisation materials on abrasion or degradation of bioreactor materials.

### Feedstocks

In traditional IB, high-quality substrates may be fed untreated, pretreated or in their ‘solid-free’ hydrolysate form to a fermenter. In the EnvB sector, in contrast, the feedstock is dictated by the need for waste treatment and pre-treatment options are often constrained by cost limits. The feedstock characteristics will affect the fermentation performance as well as the aging of equipment. For example, lignocellulosic materials contain chlorides (considered highly corrosive) and inorganic particles (grit) such as SiO_2_ and Al_2_O_3_ which contribute to mechanical wear [155]. Pre-treatment can lead to the formation and dissolution in the hydrolysate of certain chemicals such as aromatic compounds, phenolic compounds, uronic acids, and aliphatic carboxylic acids (acetic, formic and levulinic acid) [156]. Some pre-treatments involve the use of harsh chemicals such as alkalis, acids, oxidants (e.g., H_2_O_2_) and ionic liquids [156]. These chemicals might leach into the bioreactor and potentially impact the durability of materials, though they are more likely to contribute to corrosion in the pre-treatment tanks. Although ionic liquid from the azole family and those based on indolium are recommended as anti-corrosion agents [157], their possible effect on bioreactor materials has not been studied. Pre-treatment may also change the physical properties of the feedstock. These physical characteristics may directly affect the performance and durability of fermenter materials. IB/EnvB feedstocks may contain hard inert abrasive particles. Grit accumulation is a well-recognized problem in AD applications and wastewater treatment processes [158, 159], with specialised equipment designed for its removal [160]. In sewage treatment plants, suspended particles such as sand, grit, gravel, eggshells, bone chips and seeds can be found in the influent [161]. Broken glass and eggshells, which have the same chemical composition (calcium carbonate) as limestone, are common contaminants in the organic fraction of MSW. Glass and limestone are rated 15 and 55 times harder than wood in the Knoop scale [162]. Livestock manures often contain grit scraped from yards or housing areas where soil, mud and debris are tracked in by animals. Materials like sand bedding for dairy cows [163] and grit fed to poultry [164] also contribute to the mineral content of manures.

In pure culture processes such as bioethanol production, hydrolysis typically precedes fermentation, and the hydrolysate is screened and filtered to remove solids. As a result, abrasion-related damage is more likely to occur in the hydrolysis tanks than in fermenters.

Other novel carbon sources for microbial processes include plastic wastes subjected to hydrothermal liquefaction (HTL) [165–167]. The resulting liquor is mostly composed of non-corrosive inert materials such as paraffins, olefins and aromatics [167]. However, careful selection of materials for auxiliary equipment is required, particularly for the HTL unit, due to high temperature and pressure conditions and the use of solvents or catalysts [168].

While organic carbon is a key energy source for biotechnological processes, the use of inorganic carbon (IC) as feedstock is also attracting attention. CO_2_/CO streams are produced in power generation, steel production, cement production and ore extraction [169]. CO_2_ is also produced as a metabolic by-product in many bioprocesses (Table 2), though these are normally considered carbon-neutral when organic feedstocks are used. CO_2_ forms carbonic acid when it reacts with water, potentially causing corrosion to steels, a phenomenon known as sweet corrosion [170]. Therefore, material selection needs careful consideration when CO_2_ is used as substrate or produced as by-product. Autotrophic microorganisms can assimilate IC (CO_2_, CO or CH_4_) to produce organic carbon compounds [169]. For example, acetogenic bacteria can synthesise alcohols (ethanol, butanol and 2,3-butanediol) and carboxylic acids such acetic acid and lactic acid utilising inorganic gaseous carbon. These processes typically require H_2_ or light as an energy source, though some microorganisms are capable of utilising CO instead [169]. Other value-added products derived from CO_2_ streams include PHA and PHB, produced by H_2_-consuming and H_2_-producing organisms, respectively [169]. Despite its great potential, the commercial use of CO_2_/CO streams for biological conversion is still developing and is currently limited to a few companies [171]. Some proposed strategies to control CO_2_ emission involve CO_2_ capture from the atmosphere and storage [172, 173] or (bio)electrochemical transformation to hydrocarbons [174]. Besides these competing alternative applications (or rather solutions for inevitable CO_2_ emissions), CO_2_/CO biological conversion into value-added chemicals is the subject of many research endeavours in the last decade [175–177].

**Table 3:** Summary of process conditions for top biological products derived from biomass or waste as substrates

Process	Main product	Typical substrates	Commonly used microbes	Concentration	By-products	T (°C)	pH	Other conditions	Ref.
Solid state fermentation	Enzymes	Agro-industrial lignocellulosic wastes	Fungal species of *Aspergillus* and *Penicillium* genera	98 U/mL	CO_2_	25–50	5–8	Moisture: 35–90%, requires aeration or agitation	[18, 178] [19, 95, 179, 180]
Submerged Fermentation	Enzymes	Sugars, wastewater, and frying oil	Bacteria, yeast or fungal species	49,000 U/mL		30–70	4–9		[18, 19, 179, 181]
Fermentation	Lactic acid	Starch, sugar, and agro-industrial wastes	*Rhixopus* sp. and *Lactobacillus* sp.	20–110 g/L	Formic and acetic acid, ethanol, CO_2_	37–43	5–7		[11, 18, 95, 182]
Fermentation	PHA	Sugars, glycerol, starch, whey, vegetable oils, food waste, agricultural wastes and animal fat	*Pseudomonas resinovorans*, *Alcaligenes sp.* and *Bacillus Sphaericus*	N/A		15–58	5.7–7.7	Addition of yeast extract, peptone, or meat extract	[18, 95, 183]
Anaerobic digestion	Biogas (biomethane)	Animal manures, wheat straw, whey, sewage sludge and food waste	MMC	N/A	CO_2_, VFA, H_2_, NH_3_, H_2_S, carboxylic acids and alcohols	32–60	6.5–8.2	Addition of trace elements and other additives	[18, 184, 185]
Photo fermentation	Hydrogen			N/A	CO_2_, NH_3_	30–35	6.8–7.5	Illumination at 4000 lx	[186]
Biophotolysis	Hydrogen			N/A	O_2_	Ambient	Neutral	Light intensity: 30–200 (µE/m^2^/s)	[105]
Dark fermentation	Hydrogen	Food waste, agricultural residues, and sewage sludge	MMC	N/A	CO_2_, VFA	30–80	3–7	Solid concentration: 3–12% TS	[95, 105]
Microbial electrolysis cell (MEC)	Hydrogen			N/A	CO_2_	Ambient	Neutral- alkaline	Working potential of + 0.3 V	[187, 188]
Fermentation	Biosurfactants	Agro-industrial wastes, palm oil, crude glycerol, rapeseed oil, sunflower oil cake, and soybean oil	*Pseudomonas* (bacteria) and *Candida* (yeast)	Up to 61 g/L		25–37	5.5–7.5		[132, 189]
Acidogenic fermentation	VFA	Same as AD for biomethane production	MMC	Up to 60 g/L	CO_2_, CH_4_, H_2_, carboxylic acids and alcohols	15–60	4–11	Addition of methanogenic inhibitors such as 2-bromoethanesulfonate	[16, 190–193]
Fermentation	Succinic acid	Glucose, whey, and agro-industrial wastes	*Actinobacillus succinogenes Anaerobiospirillum succiniciproducens*, *Mannheimia succiniciproducens* and recombinant *Escherichia coli*	14–106 g/L	Ethanol; and acetic, formic and lactic acids	37–38	6.5–7.5		[11, 147]
Fermentation	Bioethanol	Lignocellulosic biomass and agro-indsustrial residues	*Saccharomyces cerevisiae* and *Zymomonas mobilis*	8–115 g/L	CO_2_	30–60	5.5–7.5		[11, 95]
ABE Fermentation	Biobutanol	Lignocellulosic biomass, sugarcane corn, molasses, and glycerol	*Clostridium* (e.g. *Clostridium acetobutylicim*)	Up to 20 g/L	Acetone, ethanol, butyric and acetic acids	37	4.5–5.5	Addition of yeast powder or NH_4_NO_3_	[197–201]

### Process-specific degradation agents

#### Biomethane production

Biomethane is a product of anaerobic digestion (AD) which is a MMC fermentation involving complex metabolic pathways divided in three main stages: hydrolysis, acidogenesis and methanogenesis. Corrosive elements in AD include VFA which are generated during the acidogenic step; acids and alkalis; H_2_S; and CO_2_. Acids dissociate giving up protons (H^+^) which react with electrons in metal surfaces, starting a cathodic reaction (corrosion) [202]. H_2_S forms hydro-sulphuric acid when dissolved in water and oxidises to sulphate (SO_4_^−2^) and sulphur dioxide (SO_2_) when exposed to air [202]. The latter is unlikely to occur in normal operation due to the lack of free oxygen under anaerobic conditions.

Several anti-corrosion strategies are commonly applied during operation in AD. Digesters may be subject to micro-aeration to inhibit the SRB responsible for H_2_S production and promote sulphide oxidation [203]. Other strategies to increase redox potential and inhibit H_2_S production include in-situ chemical addition such as ferrous salts to precipitate the sulphur in FeS form [185]; or oxidising chemicals such as H_2_O_2_ or potassium permanganate (KMnO_4_) [203]. Though reducing H_2_S is beneficial for improving biogas quality, minimising toxicity, eliminating odour and reducing corrosion, H_2_O_2_ is also a highly corrosive chemical which causes pitting in stainless steels [204], and could therefore adversely affect the lifespan of a digester.

Intensification methods proposed for AD systems include the addition of conductive functional materials which improve direct interspecies electron transfer, such as metal oxide nanomaterials, granular/powder activated carbon, graphite, graphene, carbon cloth, carbon nanotubes and biochar [185, 205]. While the conductive media may benefit anaerobic process performance (enhanced methanogenesis), it may also affect corrosion of exposed metallic surfaces: either mitigating attack by diverting electrons into microbial routes or, if placed at the metal/biofilm interface, unintentionally facilitating electron shuttling and localised MIC. Certain metal oxides act either as anti-corrosive/antimicrobial agents or as coating components that increase barrier properties. Finally, nanomaterials (e.g., Ag, CuO, ZnO, graphene, and polymer nanocomposite systems) are emerging as low-dose, multi-functional anti-biofilm/anti-corrosion solutions for MIC, though their use must balance efficacy with environmental stewardship and avoid creating unintended conductive pathways. Microbial electrolysis cells (MECs) [205] have also been exploited for AD/biogas production. Fouling of electrodic materials and membranes is currently a major challenge for this technology [206], and perhaps because of this, corrosion and aging of bioreactor components in MEC systems appears to have received little attention.

#### Biohydrogen production

Hydrogen is currently produced predominantly from fossil fuels by steam reforming of methane or coal gasification [169]. Hydrogen can be produced biologically via dark fermentation (an alternative pathway to AD), photo-fermentation, microbial electrolysis or biophotolysis [105].

In biological H_2_ production, there are multiple metabolic pathways which depend on the microorganisms involved. In some pathways, hydrogenase enzymes utilise electrons from ferrous sources to neutralise protons and form molecular hydrogen [169], thus accelerating corrosion. Conditions associated with failure of dark fermentation and AD processes are VFA accumulation and low pH [105], which may cause challenges for reactor materials, although exposure may only be short term until the process is rectified.

#### Bioethanol production

The production of bioethanol takes place in three main stages: substrate pre-treatment; enzymatic hydrolysis of carbohydrates; and fermentation of sugars into ethanol by microbes [95].

A probable cause of corrosion in bioethanol fermentation is the chlorides and acids derived from feedstock pre-treatments. Ethanol may also increase corrosion rates of stainless steel through pit formation [207]. There are no studies looking at corrosion in a bioethanol fermentation environment, however studies from beer fermentation might give some useful insights [208].

#### N-butanol/isobutanol production

N-butanol and isobutanol are the two isomers of butanol that can be produced during fermentation. Biobutanol is produced through acetone-butanol-ethanol (ABE) fermentation under anaerobic conditions [197], where acetone and ethanol are generated as by-products [199]. Aside from these products, ABE bioreactors might be exposed to corrosive agents originating from the pre-treatment of substrates, namely acids, alkali substances and derived inhibitors [129]. Biobutanol concentrations from wild-type organisms cannot exceed 13 g/L due to inhibition [197, 199], but with strain modification can reach up to 20 g/L [199]. No studies were identified on corrosion or aging of materials within an ABE fermentation environment.

#### Volatile fatty acids production

VFA or short chain fatty acids are C2-C5 carboxylic acids, namely acetic, propionic, butyric, isobutyric, valeric and isovaleric acids [16]. Most VFA are petrochemically derived, but recent years have seen an increase in bio-based VFA production [209]. Bio-VFA may be derived from anaerobic fermentation (AF), which is a modification of the AD metabolic pathway where methanogenesis is inhibited [16]. It has been widely suggested that, since there are multiple alternatives to biogas as a renewable energy source, the AD industries should focus on VFA production instead; however, the recovery and purification of VFA still pose a great challenge [16].

The conditions for AF are similar to AD in terms of feedstock, reactor modes, and nutrient requirements. AF and AD both require anaerobic conditions and can be operated in psychrophilic, mesophilic or thermophilic temperature ranges [16]. Operating conditions such as HRT, temperature and pH of AF can be manipulated to favour the production of targeted VFA species and inhibit methanogenesis [16]. Operating at high pH may require dosing with alkaline materials (e.g. NaOH), whereas low pH means a larger portion of the VFA could be in their free acid or protonated form. While low pH is a result of high VFA concentrations, and therefore a probable corrosive environment, the protonated fraction of the VFA is less corrosive [210]. Sodium cations have a corrosive effect on zinc and carbon steel [211]. VFA concentrations in bioreactors are typically below 60 g/L [16], and, therefore, the corrosive effects of VFAs are relatively limited.

#### Lactic acid production

Lactic acid concentrations using mutant strains can be as high as 90–110 g/L [11], but typical ranges are 20–90 g/L [18]. In a study looking at the effect of lactic acid fermentation products on corrosion of low-carbon steel, it was found that acetic acid, lactic acid and CO_2_ had a detrimental effect on the steel structure within the 120-hour duration of the experiment [212]. Ethanol acted as a corrosion inhibitor, with a corrosion rate 7 times lower than that of a sample submerged in distilled water [212]. However, distilled water is not a representative medium since carbon dioxide dissolves into it when it is in contact with air and forms carbonic acid, acidifying the water. Other studies by the same authors found that biofilms and EPS generated by *Lactobacillus* sp. act as corrosion inhibitors against 10% HCl [213] and sea water [214], respectively. Although biofilms are known as a major cause of MIC, in these studies biofilms appear to have a protective application against corrosion. However, the harsh environments of these studies (sea water and 1% HCl) do not represent the majority of the lactic acid fermentation environments. In addition, the short durations of the tests (72 and 120 h) are not representative of the time required for MIC caused by biofilm.

#### Succinic acid production

While succinic acid is typically produced from petrochemical routes by the catalytic hydrogenation of maleic anhydride [95], fermentative processes present a competitive alternative [147].

Fermentation typically takes place at mesophilic temperatures (37–38 °C) and neutral/slightly acidic pH (6.5–7.0) [195, 196]. Due to the mismatch in optimum hydrolysis and fermentation conditions, these steps are likely to take place separately, and only the hydrolysate (liquid broth) is used in the fermentation [215]. Chemicals such as Na_2_CO_3_, NaHCO_3_, Mg(OH)_2_, MgCO_3_, NaOH and NH_3_.H_2_O may be used as pH regulators [215]. Typical nitrogen sources include yeast extract, peptone, urea and corn steep liquor [215]. Succinic acid concentrations in fermentation processes range from 14 to 106 g/L [11, 147], though some studies have reported concentrations of up to 151 g/L [194, 215]. One advantage of succinic acid fermentation is that CO_2_ is not produced as a by-product, but is consumed in the reductive metabolic pathway [194], hence CO_2_ could be used as co-substrate. Despite the acidic nature of this product, succinic acid has been reported as a corrosion inhibitor in ferrous metals, though the concentrations tested are low (< 0.1 g/L) [216]. Other possible corrosive by-products from succinic fermentation are acetic acid and ethanol [194]. No studies were found relating to corrosion in the succinic acid fermentation environment.

#### Biosurfactants production

Surfactants contain components known as amphipathic motifs, the properties of which allow emulsification, foam formation, detergency and oil dispersion [132]. They can be synthetic or of biological origin. Biological surfactants (biosurfactants) are typically biodegradable, non-toxic and environmentally friendly [132]. Microorganisms that can produce biosurfactants include bacteria, yeasts, fungi and algae [132]. Biosurfactants can be classified according to molecular structure as glycolipid, lipopeptide/lipoprotein, glycoprotein, phospholipid/fatty acid, polymeric, particulate or polysaccharide-lipid complex [132], with glycolipids being the most commonly produced.

The metabolic pathway followed in the production process is dependent on the chemical composition of the feedstock [132]. Fermentation conditions are typically in the mesophilic range (25–37 °C) and slightly acidic to neutral pH (between 5.5 and 7.5) [189]. Nitrogen sources include urea, yeast extract, NaNO_3_, NH_4_NO_3_, peptone and meat extract [189]. Biosurfactant concentration may be up to 61 g/L [132]. Biosurfactant production may also take place by solid state fermentation [217], subsequently requiring an extraction process.

Biosurfactants show corrosion inhibition properties and have recently been investigated for anti-corrosion applications [218]. The inhibition effect of surfactants occurs by means of protective layer formation against corrosive substances, reducing current densities, as well as by inhibition and destruction of biofilms [218]. The primary corrosion inhibition mechanism, however, is due to biocidal effects on ‘corrosive’ microbial strains [217, 218]. Therefore, corrosion of metals does not appear to be a major concern in biosurfactant production.

#### PHA production

Bio-based plastics may have desirable properties compared to fossil-based plastics, such as biocompatibility, non-cytotoxicity, elasticity, wear resistance, and biodegradability [95]. The most researched bio-based and biodegradable plastics are PLA, PHA, and PBS [114], though PHA is the only industrially produced bio-based plastic produced through microbial processes. Biodegradation of PHA can occur naturally under both aerobic and anaerobic conditions [219]. PHA exist in different forms such as polyhydroxybutyrate (PHB), poly(3-hydroxybutyrate-co-3-hydroxyvalerate) (PHBV), poly(3-hydroxypropionate) [poly(3HP)], and more.

PHA are produced intracellularly under limited nutrient supply (low nitrogen, potassium and phosphorus), and serve as energy storage for the cell [183]; therefore, PHA are unlikely to interact with the fermenter walls or components. Yields are typically reported in terms of PHA content (%TS) [183], with the subsequent PHA extraction process involving cellular lysis and separation of the PHA from unwanted biomass and solvent [220]. In PHA production, corrosion is probably of more concern in the hydrolysis or recovery stages, due to the use of corrosive chemicals such as solvents.

#### Enzymes production

Enzymes are biocatalysts that accelerate specific reactions [95]. They are produced by all living organisms, but microorganisms present the advantages of fast growth [95]. Microbial enzymes can be produced by SSF or SmF. SSF is typically carried out by fungal cultures to produce enzymes such as cellulase, xylanase, protease, pectinase, amylase and lipase [11] as well as ligninolytic enzymes, i.e. enzymes that disrupt the lignin structure, which are divided in two sub-groups: peroxidases and laccases [221].

In SSF, temperature, pH, substrate, aeration and agitation must be controlled [11]. Reactor types that can be used for SSF include tray fermenters, rotating drums, screw reactors, stirred aerated bioreactors, rocking drum bioreactors, fluidised bed bioreactors or column fixed bed fermenters [11, 19, 178]. Tray bioreactors can be made of wood, plastic, bamboo or metal [19, 178], but metal ones must be made of stainless or coated steels to avoid rusting [178]. Packed bed reactor columns are typically made of metal or glass [178]. No relevant studies on corrosion in SSF were found. However, the high moisture content, high free oxygen availability, potential for abrasion caused by substrate mixing, and the potential for acidic by-products and formation of biofilms are causes of concern for corrosion. In contrast, because of the need for free oxygen in SmF for enzyme production, stirred tank reactors with aeration, bubble column reactor and airlift reactors are used [178].

## The role of extremophiles in industrial biotechnology

The above discussion addresses some established and emerging industrial biotechnologies, which predominantly operate under mild conditions favourable to the growth of most microbial species. Future IB/EnvB processes will be based on the utilisation of low-value mixed substrates in low-cost bioreactors, operating under non-sterile conditions which are resistant to contamination, and may involve replacement of freshwater by seawater, with use of strains capable of rapid growth [222] and enhanced biofilm formation capabilities [223].

The incorporation of extremophilic micro-organisms potentially offers significant advantages: processes utilising extremophiles exhibit enhanced resistance to contamination from other microbial species, thereby obviating the need for sterile conditions [224]. Bioreactors for processes based on extremophiles may, however, be exposed to a wider range of conditions than in conventional bioprocesses, including temperature (*T* > 75 °C for hyperthermophiles, *T* < 5 °C for psychrophiles), pH (below 5 for acidophiles, above 8.5 for alkaliphiles), or low water availability (for xerophiles), potentially in combination [224, 225]; see Table 3. Halophiles can withstand high salt concentrations (> 30 g NaCl L^−1^), making them ideal for seawater environments [224]. Even greater extremes are possible: for example, *Methanopyrus kandleri* strain 116 is capable of growing and producing methane at temperatures of 122 °C [226]; however Table 4 focuses on processes with more immediate commercial applications.

Extremophiles can be exploited to produce extremozymes and extremolytes. Extremolytes, including polyols, mannose derivatives, and amino acids, find extensive applications in the food, cosmetic, and pharmaceutical industries [227]. On the other hand, extremozymes produced by psychrophiles, halophiles, thermophiles and alkaliphiles are capable of functioning under extreme conditions, such as high salinity (4 M NaCl or KCl), elevated temperatures (up to 80 °C), and the presence of organic solvents (10–20% v/v methanol, ethanol, n-butanol or isoamyl alcohol) and ionic liquids (20–40% v/v), as well as across a broad pH spectrum (between 3.0 and 10.5) [227]. These conditions are more challenging than those commonly required for enzyme production. The development of these enzymes has become a focus in recent IB applications, particularly in the production of biofuels and other lignocellulosic biomass-derived products [225]. Notable examples of these enzymes include β-galactosidase, α-amylase and cellulase, which are utilised in the detergent, textile, food and paper industries, as well as in the saccharification of lignocellulosic waste in biorefineries [225, 227]. Extremophiles have also shown potential for lignin degradation to produce aromatic compounds [225]. Additionally, lipases and esterases play a crucial role in biodiesel production [227]. Extremophile microbes have also been successfully exploited for protease production under conditions such as high temperatures (80 °C), pH above 9 or below 3, or high salt concentration (2 M) [181]. However, only a limited range of these enzymes is currently produced on an industrial scale [227]. Extremophiles have also been successfully exploited at lab scale to produce PHAs, carboxylic acids, bioethanol, biomethane and biosurfactants [228], though the carbon source used in the majority of these studies was glucose [228].

While results are promising, further developments are required before full commercialisation of extremophile-derived products [222]. High salt concentration can result in frequent and expensive maintenance of equipment [225]. The conditions encountered in bioprocesses using extremophiles are likely to be less extreme than those already found in many common physico-chemical processes; while the bioreactor manufacturing industry is developing new polymeric materials and coatings to replace SS parts and overcome corrosion problems [225]. Commercial applications for thermophilic and psychrophilic enzymes exist in the paper, detergent, biofuel and pharmaceutical industries [229]. However, most processes that utilize extremophilic microorganisms are still in the development phase.

**Table 4:** Summary of products from extremophilic microorganisms and their associated fermentation conditions

Main product	Extreme conditions	Reference
Cellulase	pH < 4 or pH > 10 *T* > 70 °C (fungal)	[230]
Lipases	*T* < 25 °C or *T* = 50–90 °C Up to 30% NaCl pH < 5 or pH > 9	[231]
Protease	pH = 9–12 *T* < 25 °C or *T* = 50–80 °C Up to 4.5 M NaCl	[232]
PHA	0.2–5.2 M salt	[228]
Lactic acid	pH < 3	[228]
Biomethane	pH > 10	[228]
Bioethanol	pH > 10 *T* > 50 °C	[222, 228]
Succinic acid	*T* > 50 °C	[228]
N-butanol/isobutanol	*T* > 50 °C	[228]
Biosurfactants	*T* < 15 °C or *T* > 50 °C pH 2–3 13–25% NaCl Growth in hydrocarbon solutions	[233]

## Challenges in materials testing for industrial and environmental biotechnology applications

### Challenges in corrosion testing

Numerous national and international organizations, including the International Organization for Standardization (ISO), the European Committee for Standardization (EN), and the American Society for Testing and Materials (ASTM), provide standard methods for corrosion testing. These tests fall into three main categories: laboratory, pilot-scale and field tests [21]. Pilot tests replicate large-scale operating conditions on a smaller scale, while field tests expose specimens directly to real-world environments. Laboratory tests, often used for material selection and quality control, are usually short-term and include simulated atmosphere tests, immersion tests, and electrochemical methods [21]. These approaches help to assess material performance under controlled conditions and are critical for evaluating suitability in emerging IB/EnvB process environments [21].

Immersion tests assess material performance by submerging or exposing specimens to a specific environment or test solution [21, 234]. These tests accommodate a wide range of variables, including pH, temperature, halide and chemical concentrations, gas composition, flow rate, pressure and inhibitors [234]. Immersion tests are typically classified into total, partial and intermittent immersion, depending on the extent and duration of exposure [21]. Standard procedures, such as ASTM G31, G34, G35, G36, G44, G48, G61, G66, and G78 guide these tests, although this list is not exhaustive [21, 234]. These methods provide valuable insights into corrosion performance under controlled conditions and are essential for evaluating material suitability in complex process environments.

The corrosion mode, whether general/uniform, pitting, stress-corrosion cracking (SCC) or another form, depends on the type of test and the chemical composition of the test solution [21]. In immersion testing, the most commonly used method for measuring corrosion rate (CR) is weight loss [21, 234]. However, this approach may lack accuracy when corrosion is highly localized or progresses slowly. According to ASTM G31, a minimum test time (h), should be calculated using the formula [787.4 (h mm/year)/CR (mm/year)] [234]. Despite its limitations, weight loss measurement remains useful for detecting intergranular corrosion, which may not be visible at the surface [235]. Alternative evaluation methods include pit density, which measures the number of pits per mm^2^ over time, as outlined in ASTM G46 [234], and thickness loss measurements, both of which provide insights into localized corrosion behaviour [235].

Immersion tests can be combined with applied tensile stress, either constant, increasing or cyclic (as in fatigue testing) to evaluate assisted cracking mechanisms [234]. Standard tests are often tailored to specific materials, for example, enamel corrosion resistance is assessed using ISO 28,706 (1:2008; 2:2017; 3:2017; 4:2016; and 5:2010). These tests typically evaluate a material’s resistance to corrosion under defined chemical exposures and controlled conditions. However, they do not attempt to account for the complexity of microbial fermentation environments, where dynamic biological and chemical interactions can significantly influence corrosion behaviour.

MIC can also be evaluated using standard and non-standard immersion tests [234]. In the oil & gas sector, MIC resistance is typically assessed by promoting biofilm formation under controlled conditions, following standards such as ASTM E3161. Commercial bioreactors are available specifically for this purpose [236]. However, such protocols encourage the formation of biofilm by specific organisms in the absence of competing bioreactor species, and do not reflect the unique biological and chemical complexities of microbially-mediated IB/EnvB processes, especially those using waste feedstocks and MMC [237].

Electrochemical tests assess the characteristics of corrosion-related electrochemical reactions [21]. Electrochemical methods can be integrated into immersion tests [238] and are preferred over gravimetric measurements when corrosion rates are too low to detect via weight loss [21]. Key parameters assessed include [21]: open circuit potential (OCP), current, resistivity, polarization curves, linear polarization, and frequency response (electrochemical impedance spectroscopy).

Table 5 summarises the standard electrochemical tests, such as ASTM G3, G5, G61, G102, G106, G108 and G150 [21]. Electrochemical testing is most informative in IB/EnvB processes where conductive broths, fluctuating redox conditions and biofilms interact with metal surfaces, such as those that occur in anaerobic digesters and MMC fermenters, since these methods can track corrosion mechanisms in real time under process-relevant conditions. In practice, linear polarisation resistance (LPR) provides a rapid, minimally perturbative estimate of general corrosion rate by converting polarization resistance via the Stern-Geary relationship (ASTM G59/G102), which is suitable for live cultures when small overpotentials (± 20 mV) are used and assumptions (e.g., *B* value) are stated. In addition, electrochemical noise (EN) measures the corrosion potential/current fluctuations at open circuit and is well suited to online, non-invasive detection of localised/MIC activity without disturbing the microbial community (ASTM G199; also used to identify MIC signatures in field relevant tests). To translate laboratory evaluation into design decisions, a side stream loop that replicates temperature, shear and gas hold-up can house LPR/EN/OCP probes alongside mass loss coupons, with interpretation based on converging evidence. Electrochemical method limitations should be managed explicitly, estimate B parameter or Tafel slopes and address IR drop for LPR, and avoid wide potentiodynamic scans that can disrupt biofilms, hence the preference for small signal methods in live MIC studies. Together, LPR (numeric rate) and EN (early warning fingerprint of localized/MIC attack) provide a practical route to link electrochemical measurements with real operating conditions in bioreactors to allow informed maintenance and materials selection.

**Table 5:** Relevant electrochemical methods for measure of corrosion. Information obtained from [21, 23, 239]

Method	Technique brief	Advantage	Disadvantage
Linear polarisation resistance (LPR)	- Direct current - Linear current vs. potential plots - One or two electrode - Corroding interface behaves as resistor	- Can be used for the study of passivation of metals, corrosion inhibitors, and corrosion resistance of coatings - Favourable for the study of MIC	- Skewed results at high scan rates - Ineffective for localised corrosion or for highly passive materials
Potentiodynamic polarisation	- Direct current - Continuous changing potential	- Measures corrosion rate of passive metals	- Skewed results at high scan rates - Slow scan rates change the environment - Organisms in biofilm are disturbed by large potentials
Electrochemical impedance spectroscopy (EIS)	- Alternating current	- Accurate measure of resistance of polarisation - Useful for testing corrosion in resistive environments or non-conductive films - Effective at examining corrosion in coatings	- Needs analogue circuit models which may not be accurate - Requires the use of electrolytes with low conductivity - Ineffective for localised corrosion - Length of EIS measurements are shorter than fluctuations in biofilms
Electrochemical noise (EN)	- Potential or current as function of time - Fluctuations of potential at fixed current or vice versa	- Can detect many corrosion processes, including MIC - Does not disturb microbial community	- Complex interpretation of results

### Challenges in abrasion testing

Abrasiveness is an undesirable property of feedstocks, as it can damage equipment when materials are in motion. Determining abrasiveness is challenging as it depends on multiple factors such as hardness, toughness, friability, and grain structure (shape and size) [240, 241]. Among these, hardness is the most critical, and for ceramics and metals it can be measured by the Mohs, Vickers and Knoop scales [242]. The Mohs scale ranks minerals from 1 (soft like talc) to 10 (hard like diamond) [243], while in the Vickers scale, hardness can vary from 1.8 GPa (fluorite) to 60–100 GPa (diamond) [242]. The Knoop test involves pressing a diamond tip against a sample and measuring the resulting indentation [244]. In contrast, the hardness of lignocellulosic materials is rarely prioritised in feedstock testing when compared to other physico-chemical properties such as particle size, shape, moisture content, ash content, volatile content, fixed carbon, particle density, bulk density, grindability, flowability, floating index, specific surface area, porosity and moisture sorption [245–250]. For example wood, a relatively hard lignocellulosic material, has reported hardness values of 4–5 (Mohs) [251], 0.3 GPa (Vickers) [252] and 10 (Knoop) [162]. Therefore, the hardness for lignocellulosic feedstocks is expected to be at or below these values. Wood hardness is typically measured by the Janka test, which applies a steel ball and reports force in Newtons (or pounds-force) [253, 254]. Conversion charts are available to translate between the hardness scales [255]. Standard hardness tests require a ‘solid’ flat surface with a minimum surface area, which is not feasible for loose biomass like straw or husks that is fed to bioreactors or hydrolysis tanks as suspended solid particles. To overcome this, lignocellulosic biomass particles can be bonded into boards using resins or adhesives. Studies on Brinell hardness of boards made with walnut or hazelnut shells [256] revealed variability depending on the adhesive type; however, the primary limitation of the method was the inconsistency in hardness measurements across different areas of the board [256]. Areas with coarser particles exhibited greater hardness compared to those with finer particles [256].

Particle size is a key design parameter for IB solid feedstocks, as it can be controlled by grinding pre-treatments. Particle size and shape can be evaluated by 2D and 3D imaging [257]. Particle shape, influenced by both feedstock type and grinding method, is typically characterised by aspect ratios (width-to-length) [245], though this provides limited insight into the abrasive properties of the material. The wear caused by hard particles is also proportional to their velocity [39], therefore highly agitated reactors are more likely to sustain damage from abrasion. Since the particle size of feedstock materials changes during bioprocessing, as do the rheological properties of the liquid phase, this represents a further major challenge in determining the abrasive properties in real conditions.

Wear or abrasion resistance is a desired property for materials used in fermenters and equipment for handling solid-suspended particles. Abrasion resistance correlates directly with materials hardness [242]. Polymers generally exhibit low wear resistance, making it essential to consider the abrasive nature of the feedstock when selecting bioreactor materials. To enhance durability, polymer composites often incorporate fillers such as aluminium oxide (alumina) [258]. Several standardized methods exist to assess abrasion resistance. ISO 4649/DIN 53,516 measures the volume lost from a specimen after contact with an abrasive surface on a rotating drum [259]. Other widely used tests include Taber abrasion testing (ASTM D4060 and ASTM D1044), dry sand/rubber wheel (ASTM G65), and pin abrasion testing (ASTM G132) [260, 261]. Another abrasion/erosion test (ASTM G76) propels abrasive particles using compressed air [258]. These tests typically use dry silica sand and are conducted under dry/atmospheric conditions. Currently, no standard test methods exist for evaluating abrasion resistance under submerged or slurry conditions, which limits applicability to bioprocess environments.

No studies have yet investigated the wear resistance of materials specifically within bioreactor environments. However, one tribological study examined various Fe-Cr alloys (stainless steels) and a high-carbon steel for use in corn stover biomass milling [155]. Using a pin-on-disk setup in accordance with ASTM G99-17, the study submerged samples in a biomass slurry to simulate wet conditions [155]. The wear mechanisms reported under submerged conditions differed from those under dry/atmospheric conditions: while abrasion and delamination dominated dry tests, submerged stainless steel samples primarily exhibited pitting corrosion and adhesion with a moderate abrasion wear [155]. The pitting corrosion was attributed to chlorides present in the biomass slurry, whereas the high-carbon steel showed no significant pitting [155]. Interestingly, the biomass slurry acted as a lubricant, reducing solid-solid contact and thereby lowering wear volume of the submerged samples [155]. This finding highlights the need to study abrasion and corrosion together in submerged systems, as their interaction can influence materials degradation. Despite its novel insights, the study had some limitations. The mechanical wear under the submerged conditions is caused by the pin, not the biomass itself, which had been refined considerably and lacked abrasive particles such as grit. Additionally, the short test duration (45 min) may not reflect long-term corrosion performance. The study also did not take into account the possibility of MIC, which would require modifications such as a deeper liquid chamber for anaerobic conditions, microbial inoculation, adequate mixing, and nutrient additions.

### MIC monitoring and testing

In systems where biological activity is minimal or undesirable, microbial growth may pose a significant threat due to its association with MIC. Culture-based techniques [22, 26] are commonly used to detect microbial presence, but they often yield misleading results. This is primarily due to the difficulty of sampling directly from biofilms, while suspended samples may not be a true representation of the MIC-relevant microbial population [26]. In bioreactors, where microbial proliferation is intentional and essential, there is a large and sometimes diverse microorganism population, and thus culture-based tests are not reliable indicators of MIC risk.

Although no standardized tests exist for MIC resistance, accelerated tests have been developed for specific applications. These typically involve inducing biofilm formation on test specimens [238]. Gravimetric methods, surface analysis and electrochemical methods can be used to study the effect of the biofilm on corrosion [238]. Electrochemical methods such as open-circuit and redox potentials, polarisation resistance, linear polarisation, potentiodynamic techniques, EIS, scanning vibrating probe, microelectrodes, EN and dual cell techniques have been shown to give some meaningful insights into MIC, though no single method is universally applicable and each has limitation depending on the system and conditions [23].

## Bioreactor material degradation studies

Standard corrosion tests offer useful insights into material resistance to primary chemicals or products, but they fail to accurately replicate the complex characteristics found in bioreactors and fermentation environments. Real-world bioreactor studies provide a better insight by accounting for microbial activity, gaseous and liquid products, and suspended solid particles. However, the literature on corrosion performance of materials utilised within a bioreactor/fermenter environment remains limited.

One of the earliest studies, published in 1988, investigated steel corrosion in an anaerobic digester [262]. Steel specimens were submerged in different components of the plant, such as the heater, digester and sludge-storage tank for 50 days [262]. Corrosion rates were highest in the liquid phase of the digester, with lower rates reported in the headspace and at the slurry-gas interface [262]. Notably, the heater, which contained the liquid hog manure substrate, experienced significantly higher corrosion rates than the digester [262], which was attributed to the more corrosive chemical composition of the raw substrate. Although the authors suggested bio-corrosion as a contributing factor, this was not directly evaluated [262].

Another study assessed the corrosion resistance of galvanised steel (AluZinc) using an acoustic emission (AE) method to measure the hardness of the material, and compared values for a specimen before and after exposure to the bioreactor environment [263]. The conditions, substrate and duration of exposure were not reported, but the results showed a notable difference in hardness [263]. The study did not indicate whether corrosion levels were acceptable or what alternative materials could be used.

A more recent investigation used weight loss measurements to evaluate corrosion of iron-based metals in a food waste fermentation environment [264]. After just eight days, all tested metals showed rapid metal degradation or significant weight loss, particularly for intermittent submersion, suggesting these metals should be avoided in food waste fermentation [264]. This study subjected the samples to typical fermentation conditions and attributed the corrosion to the action of microbial communities; however, the corrosion mechanisms were not studied [264]. Although the feedstock was a solid substrate (10% total solids, particle size < 4 mm) [264] which typically contains grit, abrasive wear was not addressed.

These bioreactor studies were performed in the bulk, well-mixed regions of the bioreactor, and therefore do not consider ‘dead zones’ or high gradient zones, which may be more prone to mechanical damage, accumulation of abrasive or chemical materials, or corrosion due to biofilm formation.

Another long-term study examined the aging of steel (st-37), thermoplastics (polyethylene and polypropylene) and GFP over eight months in an anaerobic digester treating manure under mesophilic conditions [265]. The focus was on mechanical properties relevant to paddle-mixer applications. All materials were affected by aging, though the aging mechanisms of the thermoplastics were not discussed [265]. The GFP aged due to moisture penetration, which led to cracking, debonding and delamination of the material [265]. As expected, the steel samples suffered degradation via corrosion, mostly attributed to ammonia, salinity and organic acids [265]. The study did not discuss the type of corrosion or evaluate the possibility of MIC.

## Outstanding issues and key challenges

Earlier sections identified a range of chemical environments likely to arise in IB/EnvB applications. New biotechnologies will continue to emerge, but even those involving extremophiles are unlikely to extend this range very significantly, since biological systems typically operate within relatively narrow limits (e.g. pH 4 to 9, temperature to 75 °C). Exposure conditions associated with known metabolic processes are therefore generally still mild compared to those encountered in some common physico-chemical production stages. As noted, the harshest conditions often arise during feedstock pre-treatment or in early processing stages. In contrast, fermenter contents are typically less chemically aggressive and, in some cases, may even offer a protective effect.

The main issue with respect to bioreactor corrosion and surface degradation is thus not so much extreme or challenging new conditions, as a lack of knowledge of how these will interact with new materials being developed for the IB/EnvB market. Particular aspects of concern include increased abrasion from waste and agro-industrial feedstocks, and the potential for MIC. When combined with the use of new, cheaper, and potentially less resistant tank materials, these may be problematic for new biotechnologies, especially where these are under pressure to seek cost-effective solutions for low-margin products in competition with established fossil-based industries.

Abrasion is a key mechanism which is understudied in the context of IB/EnvB applications. Questions on how to characterise the abrasiveness of waste feedstocks or fermenter contents are rarely addressed and remain largely unresolved. Existing tests to measure hardness and abrasion resistance of materials are well established; but there is no knowledge base relating the results to exposure or impact in bioreactor systems. Existing standard abrasion tests are conducted under dry conditions, and do not account for the combined action of abrasion and corrosion in bioreactor environments, where materials are submerged in the fermenter contents or exposed in a saturated headspace.

Mixing and mass transfer are doubly significant in this context, especially when handling waste-related feedstocks. Concentration gradients and dead zones influence metabolic activity, which in turn affects local environmental conditions, as well as the fermenter broth properties [177]. Mixing also directly affects wear and abrasion of reactor surfaces. It can promote formation or removal of biofilms or corrosion products, which may be either protective or aggressive in terms of further corrosion. Advances in computational fluid dynamics can now provide a more detailed picture of reactor environments that may help to elucidate these points [266]; more ambitious goals of linking metabolic, rheological and hydrodynamic aspects across a wide range of scales, from micro-environments to bulk reactor contents, will give enhanced insights not only on process performance, but also on the causes of and factors influencing bioreactor materials corrosion and degradation [177, 266].

Current materials testing methods do not adequately reflect existing or anticipated exposure in IB/EnvB applications, so new protocols that are well-defined and robust need to be developed. More lab-based studies are needed on corrosion and degradation processes in ‘live’ bioreactor environments with real-world feedstocks, and these should be of sufficient duration for microbial effects to be established and observed. Such studies can improve our understanding of the factors and mechanisms involved; but are time-consuming and the results do not necessarily directly inform materials choices; so rapid testing methods are also essential. There is a particular need for testing methods that allow realistic comparison between current and new bioreactor materials, so that equipment suppliers and purchasers can make informed decisions on likely durability, lifetime costs and safety requirements [267]. Possible protocols for abrasion might be based e.g. on repeated standardised reciprocating or other motion in mildly abrasive fluids of known/reproducible composition, as a relatively rapid way of assessing materials. If these become common, they may also provide a way of comparing the abrasiveness of real bioreactor contents under standard conditions with that of known standard fluids; and thus offer a practical stepping stone towards understanding the effect of bioreactor conditions. There may also be scope to develop test protocols based on more realistic simulation of conditions found in bioreactor operation. One option is submerging the specimens of interest in a bioreactor environment for longer periods of time (weeks to months), although this is still uncommon in industrial practice [267].

Electrochemical techniques are particularly promising for the investigation of aging or damage to bioreactor materials, as they can provide insights into possible microbial and physico-chemical mechanisms, especially when combined with other methods. They also have limitations, such as difficulty in detecting localized corrosion. New protocols for tests that can replicate complex fermenter fluids and the presence of microbes would be valuable to enhance understanding of these systems. If used in the context of MIC, however, the application of currents and potentials may also risk altering microbial metabolism or community structure. Growing interest in the use of bioelectrochemical systems in IB/EnvB applications is contributing new insights [188], with potential for knowledge transfer between these areas. Data interpretation is likely to remain complex in all cases, but the increasing availability of AI and machine learning tools may facilitate this in future [237].

The lack of reliable, standardized methods for assessing the durability of existing and novel materials in IB/EnvB applications presents significant challenges for end users, and can place responsible, well-established material supply companies at a commercial disadvantage [267]. Such companies invest in materials innovation but are reluctant to recommend or supply products until they have robust evidence supporting their long-term performance. In contrast, suppliers offering novel products at the lower-cost end of the market may provide less rigorous guidance, either due to limited experience or a lack of commitment to thorough durability assessment.

Data collection on bioreactor materials subjected to long-term exposure in real applications should be regarded as a priority; ideally this activity should be planned from the design and commissioning phases, to ensure that access points are provided and inspection periods allowed. Challenges here include additional capital and operating costs, and discontinuities in information transfer across the cycle of design, construction, commissioning, operation and maintenance [267]. Establishing an open database of corrosion and abrasion effects from industrial systems would be another invaluable step. Given that access to bioreactors is rarely available, end of life inspection is likely to be the most promising option [267]. Records from equipment failures would be particularly useful, though in practice there are likely to be difficulties in gaining access to these for reasons of commercial confidentiality. The knowledge gained could, however, feed into the development of improved monitoring and maintenance protocols that will be of benefit to the industry and the wider public.

This review is primarily concerned with how process conditions affect bioreactor materials. However, the inverse relationship is equally important: materials can also influence the process itself due to leaching, catalytic or surface-mediated effects, and adsorption of nutrients, inhibitors or products. Failure to consider both directions may lead to unexpected performance issues, or product quality problems. The current work also focuses on structural materials used in bioreactors and vessels; this perspective overlooks a wide range of secondary and ancillary materials that are equally exposed to corrosive and microbially-active environments. The distinction is important because degradation failures in fermentation do not often originate in the vessel wall, but rather in seals, gaskets, welds, agitator shafts, sensor housings, pipework and other components. Further studies in this area are needed.

Resolving these issues is important given the potential size of markets for bio-products, and the consequent anticipated expansion in demand for tanks and equipment. While each fermentation has different conditions, there are common drivers of corrosion, and common issues in testing protocols; a common cross-sector effort to resolve them could help significantly to reduce the risks of financial loss from degradation of bioreactor materials in the emerging IB/EnvB sector.

## Conclusions and recommendations

This paper reviews the implications of current and emerging environmental/industrial biotechnologies using waste and agro-industrial feedstocks for materials selection and testing requirements of bioreactors and ancillary equipment. Based on research trends and economic projections, microbially-mediated processes are expected to target bio-based products such as methane, hydrogen, ethanol, n-butanol/isobutanol, surfactants, PHA, enzymes, and organic acids, particularly VFA, lactic acid and succinic acid. The operating conditions of these processes have been summarised to identify factors with the potential to contribute to deterioration of structural or other working components in bioreactors. While each IB/EnvB process has different conditions, there are some common corrosion drivers which can include water, chlorides, acids and alcohols, CO_2_, and H_2_S. Extrapolating from these, the review identifies a number of shortcomings and knowledge gaps regarding corrosion behaviour and materials testing protocols.

Few studies have investigated materials corrosion and degradation within bioreactor environments; and although MIC is well-documented in other sectors, its impact in fermentation systems remains under-explored. Accelerated corrosion tests (such as ASTM and ISO) are widely utilised, but generally fail to replicate bioreactor conditions in a realistic or appropriate manner. Submersion tests are useful for chemical corrosion, but typically too short to allow for microbially-influenced impacts such as biofilm formation. Electrochemical methods can provide useful insights, especially into MIC, but have difficulty in detecting localised corrosion, and are challenging in terms of data interpretation.

More representative approaches could involve development of testing protocols based on exposure to real or simulated bioreactor contents under standard conditions, complemented by longer-term submersion of specimens in actual bioreactor environments. Such studies require standardisation, however, and may still present challenges, such as accounting for behaviour in ‘dead zones’ or studying the combined effects of abrasion and corrosion. Abrasion is a key contributor to deterioration and degradation and is likely to be an increasing issue with the use of waste and related substrates, but is understudied in IB/EnvB applications.

To address some of the issues identified, new testing protocols for IB/EnvB applications should:

- Allow rapid realistic comparison between the properties of existing and novel bioreactor materials with respect to durability in such applications.
- Enable meaningful measurement of the abrasiveness of biomass feedstocks and fermenter contents.
- Provide ways to assess the resistance of materials to combined abrasion and corrosion under bioreactor conditions.

Addressing these issues will help to improve process design and material selection, reduce equipment degradation, lower capital costs, and enhance the financial viability and sustainability of IB/EnvB processes. Real-world data from bioreactors and components in long-term operation also represents a vital resource, and initiatives are needed to improve both quantity and accessibility of information.

## Data Availability

No datasets were generated or analysed during the current study.

## Appendix A

See Table 6.

**Table 6:** Methodology followed in the Scopus search of publications to generate Fig. 1. Including subject areas: Agricultural and Biological Sciences; Biochemistry, Genetics and Molecular Biology; Immunology and Microbiology; Chemical Engineering; Environmental Science; Chemistry; Engineering; Energy

Compound	No of publications 2005–2025		Search words used in article title, abstract and keywords
Lactic acid	24,303	Lactic acid fermentation	
Bioethanol	19,299	Bioethanol	
Methane	18,888	Anaerobic digestion methane	
PHA (PHB, PHBV, poly(3HP))	13,406	polyhydroxyalkanoates OR polyhydroxybutyrate OR poly 3 hydroxybutyrate AND co 3 hydroxyvalerate OR poly 3 hydroxypropionate	
Volatile fatty acids	10,412	“volatile fatty acids” fermentation	
Enzymes	6,442	Enzymes production solid state fermentation OR enzymes production submerged fermentation	
Hydrogen	6,202	Biohydrogen	
Succinic acid	2,715	succinic acid fermentation	
Formic acid	1,661	“formic acid” fermentation	
n-Butanol/isobutanol	1,080	n-butanol fermentation OR isobutanol fermentation	
Biosurfanctants	729	biosurfactants fermentation	
Itaconic acid	309	Itaconic acid fermentation	
Levulinic acid	255	Levulinic acid fermentation	
1,2-Propanediol	182	“1,2-Propanediol" fermentation	
Muconic acid	74	Muconic acid fermentation	
1,3-Butanediol	41	“1,3-butanediol” fermentation	
Glucaric acid	38	Glucaric acid fermentation	
Biomethanol	27	Biomethanol fermentation	
2,5-Furandicarboxylic acid (FDCA)	20	2,5-Furandicarboxylic acid fermentation	
3-Hydroxypropionic acid	8	3-Hydroxypropionic acid fermentation	
